# Prognostic value of coronary artery calcium scores from 1.5 mm slice reconstructions of electrocardiogram-gated computed tomography scans in asymptomatic individuals

**DOI:** 10.1038/s41598-022-11332-3

**Published:** 2022-05-03

**Authors:** Suh Young Kim, Young Joo Suh, Hye-Jeong Lee, Young Jin Kim

**Affiliations:** 1 Department of Medicine, College of Medicine, Yonsei University Graduate School, Seoul, Korea; 2 Department of Radiology, Gangneung Asan Hospital, University of Ulsan College of Medicine, Gangneung, Korea; 3 Department of Radiology, Severance Hospital, Research Institute of Radiological Science, Center for Clinical Imaging Data Science, Yonsei University College of Medicine, 50-1 Yonsei-ro, Seodaemun-gu, Seoul, 03722 Korea

**Keywords:** Cardiology, Diseases

## Abstract

It is unknown whether the thinner slice reconstruction has added value relative to 3 mm reconstructions in predicting major adverse cardiac events (MACEs). This retrospective study included 550 asymptomatic individuals who underwent cardiac CT. Coronary artery calcium (CAC) scores and severity categories were assessed from 1.5 and 3 mm scans. CAC scores obtained from 1.5 and 3 mm scans were compared using Wilcoxon signed-rank tests. Cox proportional hazard models were developed to predict MACEs based on the degree of coronary artery stenosis on coronary CT angiography and the presence of CAC on both scans. Model performances were compared using the time-dependent ROC curve and integrated area under the curve (iAUC) methods. The CAC scores obtained from 1.5 mm scans were significantly higher than those from 3 mm scans (median, interquartile range 4.5[0–71] vs. 0[0–48.4]; *p* < 0.001). Models showed no difference in predictive accuracy of the presence of CAC between 1.5 and 3 mm scans (iAUC, 0.625 vs. 0.672). In conclusion, CAC scores obtained from 1.5 mm scans are significantly higher than those from 3 mm scans, but do not provide added prognostic value relative to 3 mm scans.

## Introduction

Coronary artery calcium (CAC) scores obtained from electrocardiogram (ECG)-gated computed tomography (CT) scans have been established as strong predictors of risk for cardiac events in asymptomatic individuals^1,2^. The absence of CAC is associated with a low risk of coronary mortality and morbidity, particularly in asymptomatic individuals^3–5^. The 2010 American Heart Association cardiac CT appropriateness criteria judged CAC scoring as appropriate in asymptomatic individuals with intermediate risk for coronary heart disease (Framingham risk score, 10–20%)^6^. CAC scoring also offers guidance on interventions to prevent cardiovascular disease in asymptomatic individuals. Recently, the American College of Cardiology/American Heart Association published guidelines for measuring CAC scores in adults at intermediate risk [a 10-year atherosclerotic cardiovascular disease (ASCVD) risk range of ≥ 7.5% to < 20%] or borderline risk (a 1-year ASCVD risk of 5% to < 7.5%) when risk-based decisions for preventive statin therapy were uncertain^7^. When a CAC score is 1–99, then statin therapy is favored; when ≥ 100, it is indicated.

The standard CT scan protocol for CAC scoring is ECG-gated, non-contrast CT at a 2.5 to 3 mm slice thickness, using 120 kVp. However, with advanced CT scanners (e.g., second- or third-generation 128- or 256-detector-row dual-source instruments), image reconstruction with thinner slices (1–1.5 mm) is available without additional radiation exposure. Previous studies reported that a small amount of CAC could be depicted with higher sensitivity using thinner slice thickness reconstructions than the standard 3 mm slice thickness^8–11^. However, detecting the presence of small calcifications in thinner slices did not significantly increase the value of CAC for diagnosing the presence of obstructive stenosis^11^. Moreover, the relative prognostic value of obtaining CAC scores with thinner slice reconstruction has not been determined.

Consequently, this study was performed to evaluate whether obtaining CAC scores from 1.5 mm slice reconstructions has a better prognostic value for predicting major adverse cardiovascular events (MACEs) than using 3 mm slice reconstructions.

## Results

### Baseline clinical characteristics

Of 550 subjects, 316 (57.5%) were men, and the mean age was 59.4 ± 10.6 years. The clinical characteristics of the patient population are presented in Supplementary Table 1. The mean body mass index of the subjects was 24.0 ± 3.4 kg/m^2^ (range, 15.6 to 65.0 kg/m^2^). Diabetes mellitus, hypertension, and dyslipidemia were prevalent in 10.9%, 16.6%, and 53.5% of the study participants, respectively, and 16.7% were current smokers.

### Interreader agreement of CAC scoring

CAC scoring demonstrated high interreader agreement on both 1.5 mm and 3 mm slice thickness scans (ICC 0.99 for both; κ value for risk category 0.951 and 0.988, respectively).

### CAC scoring from 1.5 and 3 mm slice thickness scans

**Figure 1:**
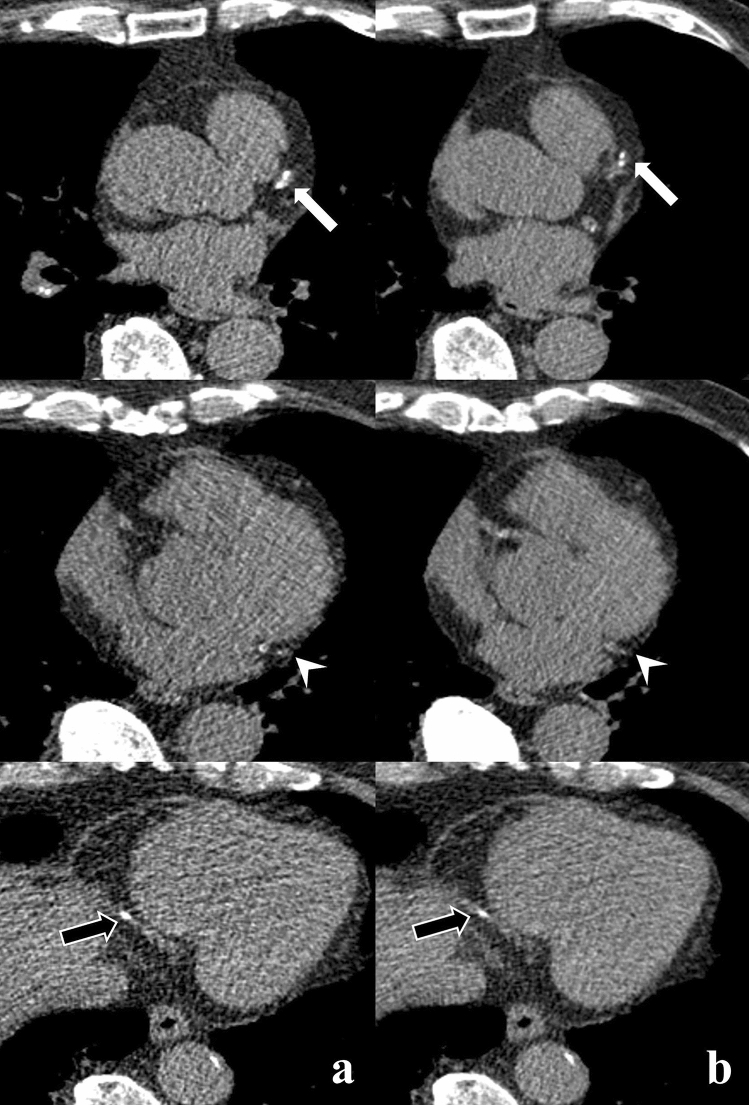
An 80-year-old male with 2-vessel CAD and moderate CAC on 1.5 mm (CAC score, 354.8) and 3 mm (CAC score, 243.0) slice thickness reconstructions. Images from a 1.5 mm slice thickness (**a**) and 3 mm slice thickness (**b**) at the corresponding scan level are shown. Calcified plaques in the left anterior descending artery (white arrow), left circumflex artery (white arrowhead), and distal right coronary artery (black arrow) are more evident in the 1.5 mm slice thickness reconstruction. The patient underwent revascularization by PCI after 272 days.

CAC scores obtained from 1.5 mm scans (IQR, 0–71; median, 4.5) were significantly higher than those from 3 mm scans (IQR, 0–48.4; median, 0, *p* < 0.001; Fig. 1). Image noise with 3 mm slice thickness reconstruction in the ascending aorta (mean ± SD, 20.1 ± 3.9) was significantly lower, as compared to 1.5 mm slice thickness reconstruction (mean ± SD, 27.4 ± 5.5, *p* < 0.001). CAC scores from 1.5 and 3 mm slice thickness scans showed excellent agreement (ICC = 0.998). The mean difference and 95% LOA between 1.5 and 3 mm slice thickness scans were 10.1 and 67.6 to − 47.5, respectively.

The agreement of CAC severity categories between 1.5 and 3 mm slice thickness scans was good (weighted κ, 0.758; 95% CI, 0.715–0.800). However, the distribution of CAC severity was significantly different between 1.5 and 3 mm slice thickness scans (*p* < 0.001; Table 1). The proportion of subjects with non-zero CAC score was significantly higher on 1.5 mm scans than on 3 mm scans (*p* < 0.001; Table 1). Among the 301 subjects with zero CAC scores from 3 mm slice thickness scans, 117 were reclassified as having non-zero CAC scores from 1.5 mm slice thickness scans. In 135 of 550 participants, more severe CAC categories were obtained from 1.5 mm scans than 3 mm scans. Two patients had more severe CAC categories from 3 mm scans than 1.5 mm scans (one subject categorized as having mild severity was re-categorized as moderate, and one subject with moderate severity was re-categorized as severe; Supplementary Fig. 1).

**Table 1:** Categorical agreement for CAC score between 1.5 mm and 3 mm slice thickness reconstruction.

	3 mm slice thickness				
	No (0)	Mild (1–100)	Moderate (101–400)	Severe (> 400)	Total (n = 550)
**1.5 mm slice thickness**					
No (0)	184	0	0	0	184
Mild (1–100)	117	137	1	0	255
Moderate (101–400)	0	14	59	1	74
Severe (> 400)	0	0	4	33	37
Total	301	151	64	34	

*CAC* Coronary artery calcium.

The associations of CAD extent with the severity of CAC score in 1.5 and 3 mm slice thickness scans are shown in Table 2. A higher degree of CAD was significantly associated with increasing CAC severity category on 1.5 and 3 mm slice thickness scans (*p* < 0.001 for all).

**Table 2:** Association of severity of coronary artery stenosis with severity of coronary artery calcification.

Severity of coronary artery stenosis	No (0)	Mild (1–100)	Moderate (101–400)	Severe (> 400)	*p* value
**Severity of coronary artery calcification on 1.5 mm slice thickness**					
No	150 (81.5)	90 (66.7)	10 (5.2)	0 (0.0)	< 0.001
Minimal (1–25%)	21 (11.4)	36 (26.7)	74 (38.1)	1 (2.7)	
Mild (26–50%)	8 (4.4)	6 (4.4)	62 (32.0)	16 (43.2)	
Significant (> 50%)					
1-vessel disease	5 (2.7)	3 (2.2)	38 (19.6)	4 (10.8)	< 0.001
2-vessel disease	0 (0.0)	0 (0.0)	9 (4.6)	9 (24.3)	
3-vessel disease	0 (0.0)	0 (0.0)	1 (0.5)	7 (18.9)	
**Severity of coronary artery calcification on 3 mm slice thickness**					
No	234 (77.7)	15 (26.8)	1 (0.6)	0 (0.0)	< 0.001
Minimal (1–25%)	49 (16.3)	30 (53.6)	51 (32.1)	2 (5.9)	
Mild (26–50%)	11 (3.7)	7 (12.5)	61 (38.4)	13 (38.2)	
Significant (> 50%)					
1-vessel disease	7 (2.3)	4 (7.1)	36 (22.6)	3 (8.8)	< 0.001
2-vessel disease	0 (0.0)	0 (0.0)	9 (5.7)	9 (26.5)	
3-vessel disease	0 (0.0)	0 (0.0)	1 (0.6)	7 (20.6)	

Data expressed as number of individuals (percentage).

### Volume and density scoring from 1.5 and 3 mm slice thickness scans

The volume scores obtained from 1.5 mm scans (IQR, 0–58.4; median, 5.39) were significantly higher than those from 3 mm scans (IQR, 0–37.8; median, 0, *p* < 0.001). The density scores obtained from 1.5 mm scans (IQR, 1.2–1.8; median, 1.61) were also significantly higher than those from 3 mm scans (IQR, 3.2–4; median, 3.71, *p* < 0.001). The volume scores from 1.5 and 3 mm slice thickness scans showed excellent agreement (ICC = 0.991, 95% CI, 0.989–0.992, *p* < 0.001). However, the density scores from 1.5 and 3 mm slice thickness scans showed poor agreement (ICC = 0.083, 95% CI, − 0.177–0.285, *p* = 0.248).

The correlation for the severity of coronary artery stenosis in relation to the volume score and density score showed ⍴ = 0.624, 0.465 for 1.5 mm (*p* < 0.001) and 0.645 and 0.35 (*p* < 0.001) for 3 mm, respectively.

### Association of clinical and CT variables with MACEs

During the mean follow-up period of 1339.8 ± 626.9 days, a total of 14 MACEs (2.6%) occurred, including one unstable angina, four nonfatal myocardial infarctions requiring hospitalization, and nine episodes of revascularizations > 90 days after the index CCTA. Subjects who experienced MACEs had higher CAC scores and volume scores from 1.5 and 3 mm slice thickness scans and more frequent obstructive CAD findings with CCTA than patients who did not experience MACEs (Table 3, *p* < 0.05 for all). Density scores were not significantly different between groups. Significant differences were observed in CAC severity categories between those who did and did not experience MACEs only in analyses of 3 mm slice thickness images (*p* = 0.026 for 3 mm and *p* = 0.075 for 1.5 mm).

**Table 3:** CAD severity, CAC score, volume score, density score. and CAC severity category for individuals with or without MACEs.

	No MACEs (n = 536)	MACE (n = 14)	*p* value
**CAD extent**			< 0.001
Normal or non-obstructive CAD	470 (87.7)	4 (26.6)	
Obstructive CAD	66 (12.3)	10 (71.4)	
1-vessel disease	44	6	
2-vessel disease	14	4	
3-vessel disease	8	0	
**CAC score (median, IQR)**			
1.5 mm slice thickness	3.8 [0, 67.7]	64.7 [6.7, 249.3]	0.019*
3 mm slice thickness	0 [0, 46.8]	40.9 [1.3, 180.3]	0.005*
**Volume score (median, IQR)**			
1.5 mm slice thickness	4.5 [0, 55.8]	50.9 [13.6. 201.2]	0.008*
3 mm slice thickness	0 [0, 35.9]	38 [1.4, 128.5]	0.006*
**Density score (median, IQR)**			
1.5 mm slice thickness	1.6 [1.2, 1.8]	1.6 [1.3, 1.8]	0.966*
3 mm slice thickness	3.7 [3.2, 4]	3.8 [3.1. 4.1]	0.661*
**CAC severity category**			
1.5 mm slice thickness			0.075
No (0)	182 (34.0)	2 (14.3)	
Mild (1–100)	249 (46.5)	6 (42.9)	
Moderate (101–400)	69 (12.9)	5 (35.7)	
Severe (> 400)	36 (6.7)	1 (7.1)	
3 mm slice thickness			0.026
No (0)	298 (55.6)	3 (21.4)	
Mild (1–100)	145 (27.1)	6 (42.9)	
Moderate (101–400)	60 (11.2)	4 (28.6)	
Severe (> 400)	33 (6.2)	1 (7.1)	

Data expressed as number (percentage).

*CAD* Coronary artery disease, *CAC* Coronary artery calcium, *IQR* Interquartile range.

*Wilcoxon’s singed rank test.

The univariable Cox regression analysis showed that characteristics of current smoking, obstructive CAD, and the presence of CAC in 3 mm scans were significantly associated with MACEs (all *p* < 0.05; Table 4). However, there was no graded relationship between increasing CAC and CAD severities and subsequent MACEs. (Supplementary Table 2).

**Table 4:** Univariable Cox regression analysis for prediction of MACE.

	Univariable analysis	
	Hazard ratio (95% CI)	*p* value
**Clinical variables**		
Age, year	1.036 (0.984–1.092)	0.177
Sex, male	2.467 (0.715–8.515)	0.153
Body mass index	1.025 (0.890–1.179)	0.735
Hypertension	1.085 (0.300–3.916)	0.901
Diabetes mellitus	0.516 (0.067–3.957)	0.524
Smoking history		
Former smoker	1.987 (0.502–7.860)	0.328
Current smoker	5.152 (1.450–18.309)	0.011
**CAD extent**		
No or non-obstructive CAD	1	Reference
Obstructive CAD	12.885 (4.089–40.606)	< 0.001
**CAC score**		
1.5 mm slice thickness		
No (0)	1	Reference
Non-zero (> 1)	3.514 (0.785–15.736)	0.100
3 mm slice thickness		
No (0)	1	Reference
Non-zero (> 1)	4.58 (1.277–16.421)	0.020
**Volume score**		
1.5 mm slice thickness	1 (0.999–1.002)	0.561
3 mm slice thickness	1 (0.998–1.002)	0.851
**Density score**		
1.5 mm slice thickness	0.992 (0.932–1.055)	0.790
3 mm slice thickness	1 (0.991–1.008)	0.911

*MACE* Major adverse cardiovascular events, *CAD* Coronary artery disease, *CAC* Coronary artery calcium.

**Figure 2:**
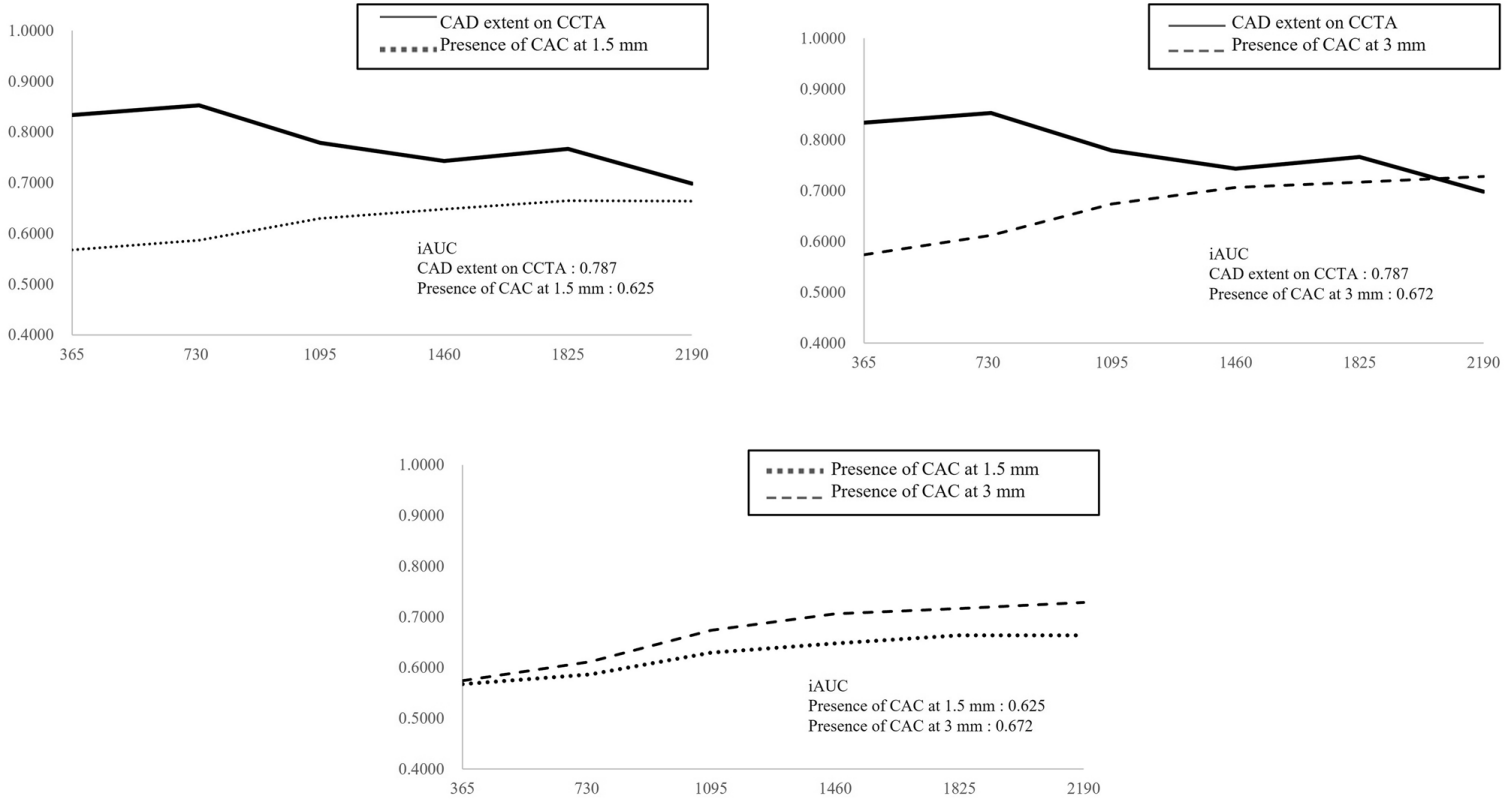
Predictive accuracy of three different models for MACEs: iAUC by follow-up time. There were no statistically significant differences among the three models. (**a**) Model 1: CAD extent. (**b**) Model 2: the presence of CAC with 1.5 mm slice thickness. (**c**) Model 3: the presence of CAC with 3 mm slice thickness.

The results of time-dependent ROC curve analyses over the entire follow-up period are shown in Fig. 2 and Supplementary Fig. 1. Values for iAUC were 0.787 for Model 1, 0.625 for Model 2, and 0.672 for Model 3.

## Discussion

Our study demonstrated that CAC scores obtained from CT scans reconstructed with a 1.5 mm slice thickness yielded were significantly higher than those obtained from 3 mm scans. Additionally, the CAC severity category was reclassified in approximately one-quarter of cases. However, CAC scores obtained from 1.5 mm scans did not add value for predicting MACEs relative to scores obtained from 3 mm scans.

Our results are consistent with previous studies that reported an increase in CAC scores with thinner slices^8–11^. In our study, 117 of 301 (38.9%) participants with negative CAC scores from 3 mm scans were reclassified as having positive CAC scores from 1.5 mm scans. Thinner slice thickness may improve the detection of calcified plaques in coronary arteries by decreasing partial volume effects, which may cause small, attenuated calcifications to be underestimated or ignored. Agatston CAC scoring depends on the size and peak voxel attenuation of a calcified lesion^12^, so detecting small voxels can result in higher scores from larger calcium sizes and higher peak attenuation.

Even though higher CAC scores are obtained from thinner CT scan reconstructions, the prognostic value of CAC scoring from thinner scans remains undetermined. Han et al. reported a prognostic significance of subtle calcified plaques on CCTA among individuals whose CAC scores were zero and suggested modifying CAC image acquisition protocols and/or scoring methods to improve the detection of subtle CAC^13^. Accordingly, we hypothesized that thinner slice imaging could detect CAC with higher sensitivity and have better prognostic value for predicting MACEs than conventional 3 mm scans.

However, our study shows that the presence of CAC in 1.5 mm scans did not improve the prediction of subsequent MACEs in asymptomatic individuals. We propose that this result can be explained in two ways. First, we included asymptomatic subjects at low risk of CAD with a low incidence rate (2.6%) for MACEs, so the prognostic value of using thin-slice CT scans could not be sufficiently demonstrated. An association between stepwise increases in the risk for subsequent MACEs and higher CAC scores and CAC severity was not clearly observed in our population, because the rate of MACEs in patients with severe CAC or 3-vessel disease was low or zero, in contrast to previous studies^1,14–17^. Therefore, we chose binary variables to categorize CAC and CAD severities (absence vs. presence of CAC and no CAD or non-obstructive CAD vs. obstructive CAD) for the prediction models, and the presence of CAC and obstructive CAD were significantly associated with a higher risk of MACEs in the univariable analysis. Second, thin‐slice reconstruction detects small calcifications better but also causes more image noise, which may bias the detection of CAC. Moreover, it is not always feasible to distinguish small calcified lesions from spurious lesions caused by image noise on non-contrast examination, even when using CCTA. Therefore, excessive image noise can mimic small calcified lesions, increasing false-positive detections that increase the CAC score.

Our study results may suggest that adjusting reconstruction parameters such as thinner slice thickness to detect small calcified lesions may not be necessary because the prognostic significance is unclear. However, further study is necessary to investigate whether 1.5 mm slice thicknesses provide added prognostic value in various populations, such as symptomatic individuals. In addition, our data increase our understanding of the clinical significance of incidentally detected CAC in non-ECG gated CT scans, where reconstruction at slice thicknesses of 1–1.5 mm is commonly applied. A recent meta-analysis reported that the agreement of CAC severities assessed by ECG-gated and non-ECG-gated CT scans was excellent; however, scan acquisition and reconstruction parameters, such as slice thickness and reconstruction kernel, could significantly affect the results^18^. Moreover, the prognostic significance of CAC detected on chest CT scans for lung cancer screening may differ according to the slice thickness, and CAC observed in thinner slice data may not have a better prognostic value, as shown in a previous study and our results^19^.

Interestingly, the CAC score values in two participants were higher in 3 mm reconstruction images than 1.5 mm scans (Supplementary Fig. 2). One reason could be because the thinner slice reconstructions resulted in better spatial resolution (smaller voxels) that reduced the partial volume effect and decreased the overestimation of calcified plaques. We hypothesize that increasing the CT image noise lowers the CAC scores in these cases. Although image noise can mimic small calcified lesions, increasing image noise can also decrease the CAC score because higher noise means that fewer voxels will be above the 130 HU threshold because of the higher variability in the values^20^.

There are limitations to this study. First, this study was conducted at a single center with asymptomatic individuals. The retrospective nature of this study is also associated with selection bias. Therefore, the results of this study may not be generalizable to all patients with CAD. Second, the overall incidence of MACEs during the follow-up period was low (14/550, 2.6%) and driven mainly by revascularization (9/14, 64.3%). Consequently, the ability to compare the predictive accuracy for MACEs among the three models was limited. Additional studies are needed to evaluate the prognostic value of CAC scoring from thinner slice reconstructions in symptomatic populations with higher rates of MACEs.

## Materials and methods

### Study population

This retrospective study has been approaved by the Institutional Review Board of Severance Hospital, Yonsei University Health System, Seoul, Korea (4–2021-0785). The requirement for informed consent was waived. The study was conducted in accordance with the Declaration of Helsinki, and the protocol was approved by the Ethics Committee of Severance Hospital.

We retrospectively enrolled 818 asymptomatic individuals who consecutively underwent cardiac CT for health check-ups at our institution between September 2013 and December 2018. In our institution, images for CAC scoring are produced from cardiac CT scans conducted at health check-ups and reconstructed with two slice thicknesses: 1.5 and 3 mm. Subjects were excluded if they had a prior history of percutaneous coronary intervention (PCI) or coronary artery bypass grafting (CABG) (n = 27), did not have a complete set of 1.5 and 3 mm reconstructions (n = 123), or had a follow-up interval of less than one year (n = 118). A total of 550 individuals [mean age, 59.4 ± 10.6 years (range, 23–84); 316 men, 234 women] were included in the analysis.

### CT image acquisition

All cardiac CT scans were performed with second- or third-generation 128- or 256-detector-row dual-source CT scanners (SOMATOM Definition Flash or SOMATOM Definition Force, Siemens Healthineers, Forchheim, Germany). The image acquisition protocol used when obtaining each scan was the one recommended by the Society of Cardiovascular Computed Tomography^6^. Non-contrast CT images were acquired with prospective ECG-gated sequential scanning with a tube voltage of 120 kV and a tube current of 50 mAs for CAC scoring. The scan range extends from the angle of the carina to below the cardiac apex. A complete cardiac non-contrast coronary artery image was acquired during a single inspiration and breath-hold at 70% or 35% of the R-R interval, depending on the heart rate. Image reconstruction was performed using a medium sharp kernel (B35f.), specifically designed to enhance calcification detection. The whole volume was reconstructed in non-overlapping data sets of 1.5 mm and 3 mm slice thickness from the acquired raw data. Contrast-enhanced coronary computed tomography angiography (CCTA) was performed during an inspiratory breath-hold with the use of prospective ECG gating. If a patient’s resting heart rate was ≥ 65 beats/min, oral metoprolol was administered 30 min before the CCTA examination. Sublingual nitroglycerin (0.3–0.6 mg) was administered immediately before contrast injection for maximal vasodilatation.

### CT image analysis

CT image analysis was performed by two radiologists (Y.J.S and S.Y.K) with 13 and 3 years of experience in cardiac CT, respectively. On non-contrast-enhanced calcium scoring scans, CAC scores were measured on 1.5 and 3 mm slice images using the Agatston method, which defines the calcific lesion on CT with a threshold of 130 Hounsfield units (HU) and an area ≥ 1 mm^2^^12^. CAC scores were evaluated using commercial software (AVIEW CAC, Coreline Soft, Seoul, Korea). Firstly, coronary lesions with attenuation > 130 HU and area ≥ 1 mm^2^ were automatically color-coded. Secondly, regions of interest showing calcium attenuation were manually selected. Lastly, the total CAC score was calculated as the sum of the individual lesion scores. We classified the results from each subject into four categories based on CAC score: none (score = 0), mild (scroe 1–100), moderate (score 101–400), and severe (score > 400)^21^. In addition to CAC score, a volume score was also calculated on 1.5 and 3 mm datasets. We used a formula using CAC scores and the volume scores to create CAC density score. The formula was: CAC score/area score = density score^22^.To evaluate interreader agreement of manual CAC scoring, 100 (18.2%) CT scans were randomly selected for 1.5 and 3 mm thickness values, with even distribution over the CAC severity categories (25 cases in each category).

Image noise was measured on 1.5 and 3 mm slice images, which was defined as the standard deviation (SD) of the measured pixel values in HU within circular ROIs in the ascending aorta on slice image at level of left main coronary artery. The degree of coronary luminal stenosis was classified by the maximal luminal diameter stenosis observed on any plane of CCTA. Stenosis was graded as minimal (≤ 25%), mild (26–50%), or significant (> 50%)^23,24^.

### Clinical outcome

Clinical follow-up data were collected by reviewing electronic medical records to identify MACEs: cardiac death, nonfatal myocardial infarction, unstable angina requiring hospitalization, and revascularization either by PCI or CABG > 90 days from the index CCTA. The dates of MACE occurrence and last follow-up visit were recorded.

### Statistical analysis

The mean, SD, and median values were calculated for CAC scores, volume scores, and density scores obtained with the 1.5 and 3 mm datasets. The Wilcoxon signed ranks test was applied to determine the statistical significance of differences between the CAC scores, volume socres, density cores, and image noise on 1.5 and 3 mm slice thicknesses. Interreader agreement of CAC scores and the category of CAC severity was evaluated using the intraclass correlation coefficient (ICC) and weighted κ statistics. The agreement of CAC scores between the 1.5 and 3 mm reconstructions was evaluated using the ICC and Bland–Altman analysis with a 95% limit of agreement (LOA). The agreement of the category of CAC severity between the two slice thicknesses was assessed using weighted κ statistics. The correlation between the volume score, density score and the severity of coronary artery stenosis were assessed with Spearman’s rank correlation coefficient (⍴). When comparing the groups with and without MACEs, chi-square or McNemar tests were used for categorical variables, and the Mann–Whitney U test was used for continuous variables. Cox regression analyses were used to identify prognostic factors of MACEs using hazard ratios (HRs) and 95% confidence intervals (CIs). We developed three models to assess the added value of 1.5 mm slice thickness CAC data in risk prediction. Model 1 includes the coronary artery disease (CAD) extent on CCTA (no or non-obstructive CAD vs. obstructive CAD). Model 2 includes the presence of CAC from 1.5 mm slice thickness images (absence vs. presence of CAC). Model 3 includes the presence of CAC from 3 mm slice thickness images (absence vs. presence of CAC). The discriminatory function of each model was evaluated using the time-dependent receiver operating characteristic (ROC) curve method, and the predictive accuracy of the models was calculated using the integrated area under the curve (iAUC). The iAUC is a weighted average of the AUC across a follow-up period, and it measures the predictive prognosis of a model during follow-up. A higher iAUC indicates a better predictive prognosis. A *p* value of < 0.05 was considered to be statistically significant. All analyses were performed with SAS (version 9.2, SAS Institute Inc., Cary, NC, USA).

## Conclusion

In conclusion, CT scans reconstructed at a 1.5 mm slice thickness scan can detect small CAC with higher sensitively than conventional 3 mm scans. However, the presence of CAC in 1.5 mm scans has no added value for predicting subsequent MACEs in asymptomatic individuals.

## Supplementary Information

Supplementary Information 1.

Supplementary Information 2.

Supplementary Information 3.

## Data Availability

The data set analysed during the current study are not publicly available due to medical confidentiality but are available from the first author on reasonable request summarized form pending the approval of the IRB.
