# Supplementary material for: Prognostic value of coronary artery calcium scores from 1.5 mm slice reconstructions of electrocardiogram-gated computed tomography scans in asymptomatic individuals
Source: Sci Rep. 2022 May 3;12:7198. doi: 10.1038/s41598-022-11332-3 (PMC9064982; doi:10.1038/s41598-022-11332-3)
Supplement: Supplementary file 1 — Supplementary Information 1. [file 41598_2022_11332_MOESM1_ESM.docx]

**Prognostic Value of Obtaining Coronary Artery Calcium Scores from 1.5 mm Slice Reconstructions of Electrocardiogram-gated Computed Tomography Scans for Predicting Cardiovascular Risk in Asymptomatic Individuals**

Suh Young Kim^a,b^, Young Joo Suh^c^, Hye-Jeong Lee^c^, Young Jin Kim^c^

^a^ Department of Medicine, Yonsei University Graduate School, College of Medicine, Seoul, Korea.

^b^Department of Radiology, Gangneung Asan Hospital, University of Ulsan College of Medicine, Gangneung, Korea.

^c^Department of Radiology, Severance Hospital, Research Institute of Radiological Science, Center for Clinical Imaging Data Science, Yonsei University College of Medicine, Seoul, Korea.

**Address for correspondence:**

**Young Joo Suh, MD, PhD**

Department of Radiology, Severance Hospital, Research Institute of Radiological Science, Center for Clinical Imaging Data Science, Yonsei University College of Medicine, 50-1 Yonsei-ro, Seodaemun-gu, Seoul 03722, Korea

Tel: 82-2-2228-7400; Fax: 82-2-2227-8337

E-mail: [rongzusuh@gmail.com](mailto:rongzusuh@gmail.com)

**Supplementary table 1. Baseline demographic and clinical characteristics of the study population**

| **Variables** | **Patients (n=550)** |
| --- | --- |
| Age | 59.4 ± 10.6 |
| Male *n* (%) | 316 (57.5) |
| Body mass index (kg/m2) | 24.0 ± 3.4 |
| Hypertension *n* (%) | 91 (16.6) |
| Diabetes mellitus *n* (%) | 60 (10.9) |
| Dyslipidemia *n* (%) | 294 (53.5) |
| Systolic BP (mmHg) | 123.6 ± 14.9 |
| Smoke |  |
| Never smoker *n*(%) | 306 (55.6) |
| Former smoker *n*(%) | 152 (27.6) |
| Current smoker *n*(%) | 92 (16.7) |
| Pack-years of smoking (n=38) | 3.4 ± 1.9 |
| Total cholesterol (mg/dL) | 192.9 ± 38.9 |
| HDL cholesterol (mg/dL) | 113.4 ± 34.5 |
| LDL cholesterol (mg/dL) | 52.6 ± 13.7 |
| MACE | 14 (2.6) |
| Myocardial infarction *n*(%) | 4 (28.6) |
| Unstable angina *n*(%) | 1 (7.1) |
| Late PCI *n*(%) | 9 (64.3) |
| Interval(d) mean STD | 1339.8 ± 626.9 |
| Interval(d) median IQR | 1255.5 [789, 1848] |
| Death *n*(%) | 2 (0.4) |

Data expressed as number (percentage) or mean ± standard deviation

Abbreviations: HDL, high-density lipoprotein; LDL, low-density lipoprotein; MACE, major adverse cardiovascular events; PCI, percutaneous coronary intervention; STD, standard deviation; IQR, interquartile range

**Supplementary table 2. Univariable Cox regression analysis for prediction of MACE**

|  | **Univariable analysis** | |
| --- | --- | --- |
|  |  |  |
|  | **Hazard ratio (95% CI)** | ***p* value** |
| **CAD extent** |  |  |
| No or non-obstructive CAD | 1 | Reference |
| 1-vessel disease | 12.985 (4.089-40.606) | < 0.001 |
| 2-vessel disease | 22.580(5.548-91.898) | < 0.001 |
| 3-vessel disease | 4.163(0.178-97.306) | 0.375 |
| **CAC score** |  |  |
| 1.5 mm slice thickness |  |  |
| No (0) | 1 | Reference |
| Mild(1-100) | 2.302(0.481-11.012) | 0.296 |
| Moderate(101-400) | 5.902(1.193-29.202) | 0.030 |
| Severe(>400) | 2.803(0.321-24.47) | 0.351 |
| 3 mm slice thickness |  |  |
| No (0) | 1 | Reference |
| Mild(1-100) | 4.135(1.030-16.604) | 0.045 |
| Moderate(101-400) | 5.712(1.282-25.462) | 0.022 |
| Severe(>400) | 3.377(0.434-26.253) | 0.245 |

Abbreviations: MACE, major adverse cardiovascular events; CAD, coronary artery disease; CAC, coronary artery calcium

**Supplementary table legends**

**Supplementary Table 1.** Baseline demographics and clinical characteristics of the study population

**Supplementary Table 2.** Univariable Cox regression analysis for predicting MACEs

**Supplementary figure legend**

**Supplementary Fig. 1.** Time-dependent ROC curve analyses of prediction models for 1-, 3-, and 5-year MACE. ROC curves of clinical parameters model for 1-, 3-, and 5-year.

^a^Model 1: CAD extent.

^b^Model 2: the presence of CAC with 1.5 mm slice thickness

^c^Model 3: the presence of CAC with 3 mm slice thickness

**Supplementary Fig. 2.** A 73-year-old male with 1-vessel CAD, a moderate CAC score on a 1.5 mm (CAC score, 376.5), and severe CAC on a 3 mm (CAC score, 482.0) slice thickness reconstructions. Images with (a) 1.5 mm slice thickness and (b) 3 mm slice thickness at the corresponding scan level are shown. Large calcified plaques in the left main coronary artery (white arrow) and proximal left anterior descending artery (black arrow) are more clearly depicted with a 3 mm slice thickness reconstruction. In contrast, a small calcified plaque in the proximal left circumflex artery (white arrowhead) is more apparent with a 1.5 mm slice thickness reconstruction.
