# Supplementary figures and images for: Prognostic value of coronary artery calcium scores from 1.5 mm slice reconstructions of electrocardiogram-gated computed tomography scans in asymptomatic individuals
Source: Sci Rep. 2022 May 3;12:7198. doi: 10.1038/s41598-022-11332-3 (PMC9064982; doi:10.1038/s41598-022-11332-3)

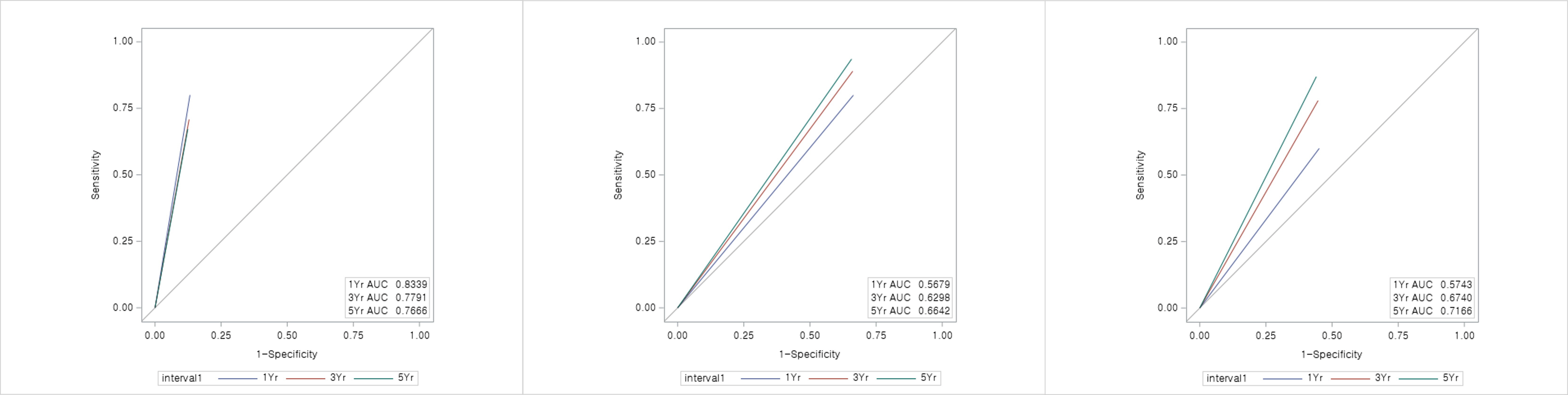

Supplement: Supplementary file 2 — Supplementary Information 2. [file 41598_2022_11332_MOESM2_ESM.jpg]
